# Data quality and associated factors of routine health information system among health centers of West Gojjam Zone, northwest Ethiopia, 2021

**DOI:** 10.3389/frhs.2023.1059611

**Published:** 2023-03-24

**Authors:** Afework Chekol, Asmamaw Ketemaw, Addisu Endale, Abiot Aschale, Bekalu Endalew, Mulusew Andualem Asemahagn

**Affiliations:** ^1^ Department of Nursing, Bahir Dar Health Sciences College, Bahir Dar, Ethiopia; ^2^ School of Public Health, College of Medicine and Health Sciences, Bahir Dar University, Bahir Dar, Ethiopia; ^3^ Department of Public Health, College of Health Sciences, Debre Markos University, Debre Markos, Ethiopia

**Keywords:** data quality, routine health information system, West Gojjam, quality, health information

## Abstract

**Background:**

Data quality is a multidimensional term that includes accuracy, precision, completeness, timeliness, integrity, and confidentiality. The quality of data generated by a routine health information system (RHIS) is still very poor in low- and middle-income countries. There is a paucity of studies as to what determines data quality in health facilities in the study area. Therefore, the aim of the present study was to assess the magnitude of the quality of routine health information system data and its determinants among health centers.

**Methods:**

A facility-based quantitative study design triangulated by the qualitative method was conducted. A total of 314 health professionals from 32 health centers were selected using a simple random sampling procedure. Data were gathered using a standardized checklist, interviewer-administered questionnaires, and key informant interview guidelines. Descriptive statistics were used to describe variables and binary logistic regression was used to identify factors associated with data quality using STATA version 14. Variables with *p*-value <0.25 in the bivariate analysis were entered to a multivariable logistic regression analysis. *P*-values <0.05 at 95% confidence intervals (CI) were taken to be statistically significant. A manual analysis was conducted for the qualitative data collected from purposively selected key informants.

**Results:**

The study found that the overall data quality at the health centers of West Gojjam Zone was 74% (95% CI 68–78). The complexity of the routine health information system format [adjusted odds ratio (AOR) 3.8; 95% CI 1.7–8.5], problem-solving skills for RHIS tasks (AOR 2.8; 95% CI 1.2–6.4), and knowing duties, roles, and responsibilities were significantly associated with data quality (AOR 12; 95% CI 5.6–25.8), and lack of human resources, poor feedback mechanisms, delay in completing data records, lack of data use, and inadequate training on health information systems were barriers affecting data quality.

**Conclusions:**

The level of data quality among public health centers in the Amhara region was lower than expected at the national level.

## Introduction

### Background

Data quality is a multidimensional term that includes accuracy, precision, completeness, timeliness, integrity, and confidentiality (1). Quality data represent what their official source intended or defined, are objective, unbiased, and adhere to established standards (2). The aim of data quality is to guarantee that data are accurate, timely, and consistent enough for the organization to make sound decisions (3).

The routine health information system (RHIS) is one of the six components of a health system that is responsible for the generation and utilization of data for various purposes (4). It also serves as a framework for all areas of the health system's decision-making, including policy development and implementation, governance and regulation, health research, human resource development, health education and training, service delivery, and support (5, 6). The goal of a health information system is to generate high-quality health data on a regular basis (7).

Since 2008, Ethiopia's RHIS has gathered and provided fundamental monitorable indicators that may be utilized to improve healthcare delivery, and the RHIS has been proven to be an invaluable tool for tracking and revising policy implementation and resource allocation (8). For health decision-making, the requirement for organized, accessible, timely, and reliable data is becoming a major problem. The Ethiopian Federal Ministry of Health has responded by reforming and redesigning the national RHIS. The reform has taken significant measures to address a lack of routine health data, which has hindered the quality of care, planning, and management systems, as well as decision-making (9).

The District Health Information Software (DHIS) is an open-source software platform that is utilized in over 60 countries for the data reporting, analysis, and dissemination for all health-related initiatives (10). RHIS is the primary information system of Ethiopia's “One plan, one budget, and one report” policy (11). As a result, the need for an information revolution was identified as one of the four transformation goals in the health sector transformation plan, which includes development on the two methodologies from data collection to decision-making. The information revolution is concerned not just with technological advancements but also with cultural shifts and attitudes toward information (9, 12).

In healthcare planning, management, and decision-making, having data that are correct, complete, and delivered on time is crucial. However, data quality is frequently evaluated as part of an RHIS efficacy or performance; yet, data quality assessment is sometimes overlooked within these scopes. This could result in a lack of understanding of data management and data quality awareness (13).

In the global health system, the quality of data created by routine RHIS in low- and middle-income countries is still quite poor (14). In India, Nepal, and Pakistan, studies show that the overall health data quality was much below the national standard (15–17).

In many African countries, data quality was found to be in the range of 34%–72% (18). According to the DHIS2, the performance report has improved across all districts, with an average of 68%, implying that only 12% is left to reach the national objective of 80% (19, 20). Ethiopia's routine health information system found that data quality is below the national average of 80%. In addition, data management and decision-making were lacking at the lower levels of the health system, and data quality assurance, feedback mechanisms, lack of accuracy, timeliness, and completeness of RHIS reporting remain a weakness. Such delays contribute to the challenge of using data as the basis for informed decision-making in healthcare planning and management (21, 22).

In Ethiopia, RHIS data quality and information use showed that the content completeness, reporting timeliness, and accuracy were 39%, 73%, and 76%, respectively (21). In another study that was conducted at Dire Dawa, the overall data quality in the unit or department was found to be 75.3% (22). Previous evidence in Ethiopia, including the South Nation Nationality People Region, suggests that the level of data quality was recorded as below the national threshold (18, 22, 23).

This evidence shows that there is a paucity of studies as to what determines data quality in health facilities in the study area and that little was known about the barriers that affect the quality of routine health information system data in public health facilities in Ethiopia. Therefore, the general aim of the present study was to assess data quality and its associated factors in the routine health information system among health centers of West Gojjam Zone, northwest Ethiopia, between September and October 2021. The specific objectives were to determine the level of data quality in the routine health information system, to identify factors affecting data quality in the routine health information system, and to explore the barriers of data quality in the routine health information system among health centers in West Gojjam Zone, northwest Ethiopia in 2021.

### Conceptual framework

**Figure 1:**
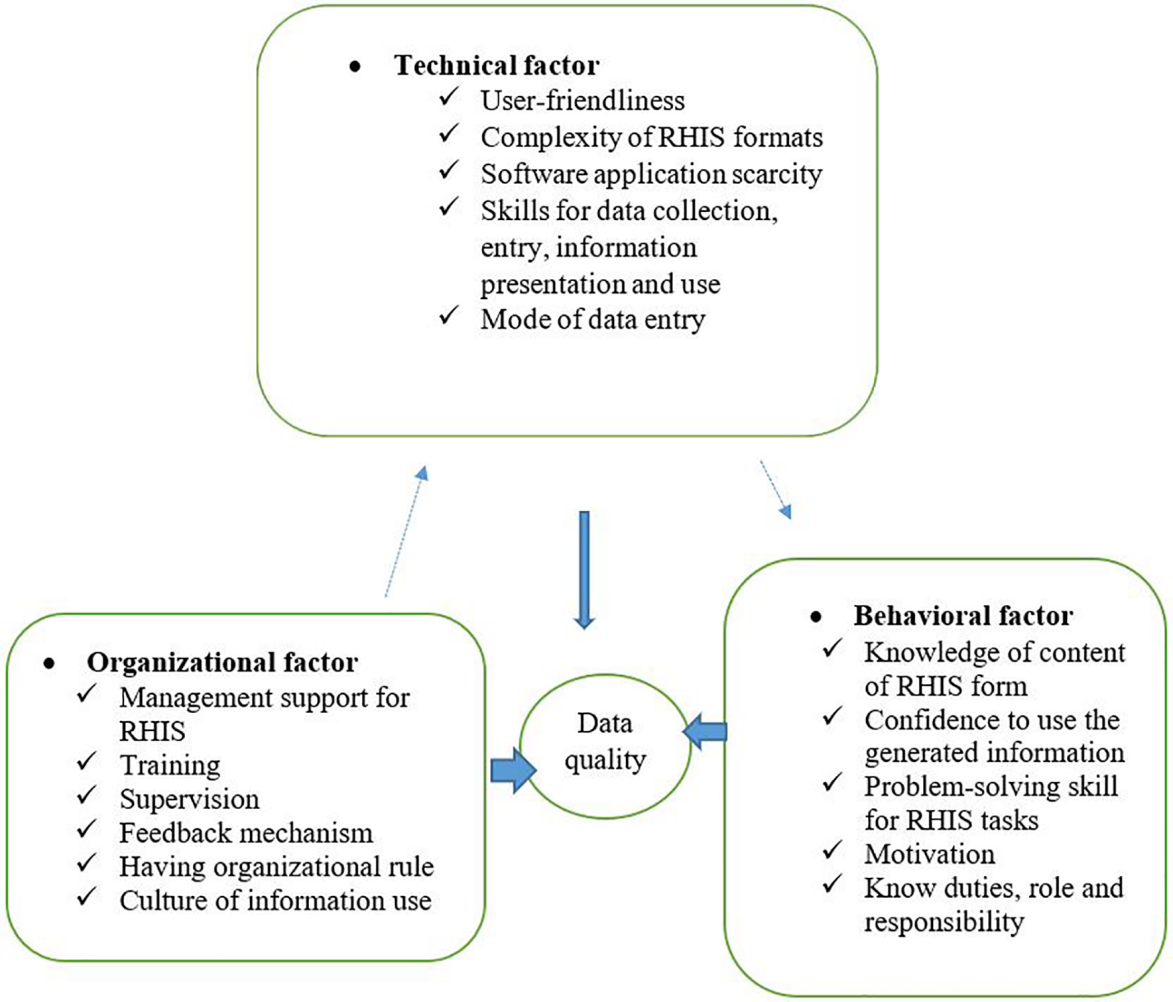
Conceptual framework of data quality and associated factors in RHIS among West Gojjam Zone, northwest Ethiopia, 2021.

The technical, behavioral, and organizational factors had a direct relationship with data quality, as indicated below (Figure 1). In addition, they show that there is a relationship between the technical, organizational, and behavioral factors. According to previous studies, those factors affected data quality. The conceptual framework is adapted from the WHO PRISM framework and other different studies (18, 24, 25).

## Methodology

### Study design

The study was conducted using a mixed-methods approach. There are approximately four major types of mixed-study designs. These are the explanatory sequential, triangulated, embedded, and exploratory sequential designs (26). The authors used the explanatory design (also known as the explanatory sequential design), which is a two-phase mixed-methods design. The rationale for this approach is that the quantitative data and results provide a general picture of the research problem; more analysis, specifically through qualitative data collection, is needed to refine, extend, or explain the general picture. The authors started with the collection and analysis of quantitative (numeric) data using a structured checklist and interviewer administered questionnaire. This first phase was followed by the subsequent collection and analysis of qualitative (text) data using the key informant interview guide, which contains open-ended questions. The second, qualitative phase of the study was designed so that it followed from the results of the first quantitative phase. Because the design began quantitatively, investigators typically placed greater emphasis on the quantitative methods than the qualitative methods. The authors started with a facility-based quantitative study and identified statistically significant differences and anomalous results. They then followed up these results with an in-depth qualitative study to explain why these results occurred.

The study was conducted at selected public health centers in the districts of West Gojjam Zone between 1 September and 30 October 2021.

### Study area

The study was conducted in West Gojjam Zone, in Amhara National Regional State of Ethiopia. It comprises 16 districts, two town administrations, and 404 kebeles. Its capital city is Finote Selam town, which is located 524 km northwest of Addis Ababa. It is bordered on the south by the Abay River, which separates it from the Oromia and Benishangul-Gumuz Regions, on the west by Awi Zone, on the north and northwest by North Gondar and South Gondar, and on the east by East Gojjam. The population is approximately 2,758,000 (1,393,197 males, 1,365,609 females). The zone has one general hospital, six primary hospitals, 108 health centers, and 404 health posts. It has 3,175 health professionals from different disciplines (26).

### Study population

The participants included all randomly selected health professionals who were working at the selected public health centers in the districts of West Gojjam Zone.

### Inclusion criteria

All public health centers that are available in the West Gojjam Zone and implemented RHIS for more than 6 months were considered to be study participants.

### Sample size determination

The sample size was calculated using the single population proportion formula based on the following assumptions: the magnitude of the data quality of routine health information system among departments in public health facilities of Dire Dawa (75.3%) (22); the desired degree of precision was 5%; 95% CI; and using a contingency of 10% for non-respondents. The final sample size was 314. The sample size was determined as follows:

$$n=\frac{Z_{a/2}^{2}p(1-p)}{d^{2}}$$

where *Z* is the standard score corresponding 95% confidence level, *p* is the magnitude of the data quality of the routine health information system among departments in the public health facilities of Dire Dawa, *D* is the margin of sampling error, and *n* is the number of the sample, as follows:

$$n=\frac{{\left(1.96\right)}^{2}(0.753)(0.247)}{{\left(0.05\right)}^{2}}+10\text{\%},\;\mathrm{non}\text{-}\mathrm{response}\;\mathrm{rate}=314$$

### Sampling procedures

The WHO recommendation for a health facility assessment considers 10%–50% of all available facilities as a representative sample. Thus, out of all 108 health centers in the zone, 30% were selected (27). A total of 32 health centers were selected randomly, of which 314 respondents were proportionally drawn. Then, health professionals who were involved in RHIS activities at each health center were selected randomly. For the qualitative method, eight participants were selected using the purposive sampling technique (head of the health center and health information technicians (HIT)) for key informant interviews (KIIs).

## Method of data collection and analysis

### Data collection tools and procedures

#### For accuracy dimensions

Based on the use of national RHIS information and the data quality manual, seven to nine data elements from each health center is satisfactory to assess data accuracy (27). Data elements were selected randomly from top priority indicators at the national level. Therefore, the seven data elements from the 32 selected health centers were verified. The documents for data collection covering 2 months were reviewed to check the consistency of selected data elements by random selection of the months September and October. The accuracy of data elements was determined by the accuracy ratio (recounted data from the source document or registrations over reported data to the next level) for the respective data element.

#### For completeness and timeliness

Completeness was assessed by the proportion of filled data elements of report content and registration content pertaining to the selected months. A tolerance level of ≥85% was used in grading the health centers, which meant that each health center expected to complete at least 85% of data elements on report content and registration content. All data elements of the 2 months of RHIS reports were reviewed to assess the content completeness of the reports.

#### Timeliness

Timeliness was assessed as a report submission within the accepted time period through observing the reporting date on the reporting form of two randomly selected monthly reports. A tolerance of ≥85% was used in grading the health centers.

Quantitative data were collected using a structured checklist and interviewer-based administered questionnaires that were adapted from the PRISM assessment tools (18, 28). The tool includes checklists to measure the accuracy, completeness, and timeliness of the data quality. It also includes the background information of the respondents and organizational, behavioral, and technical determinants of data quality in the health centers.

Qualitative data were collected using the KII guide, which contains open-ended questions. The interviews were conducted face-to-face and were recorded for an average of 30 min per participant using a tape recorder and notes were taken by a note-taker. The principal investigator facilitated the interview process. Three health professionals who were experienced and had training in RHIS-related tasks were recruited for data collection. The qualitative data collector had a master's degree in public health and had previously taken a qualitative research course, while the two health professionals who collected the quantitative data each hold a BSc degree in Nursing.

## Study variables

### Dependent variables

The dependent variable was data quality (yes/no).

### Independent variables

The technical factors were as follows: user-friendliness; complexity of RHIS formats; scarcity of software applications; training for data entry; method of data entry; lack of skill in data collection, analysis, information, presentation, and use.

The individual/behavioral factors were as follows: knowledge of content of RHIS formats; confidence to use the generated information; problem-solving skills for RHIS tasks; motivation; and aware of duties, roles, and responsibilities.

The organizational factors were as follows: management support for RHIS; training; supervision; feedback; organizational rules; and presence of culture when using information.

### Data quality management/assurance

For the quantitative study, a pretest was conducted by taking 5% of the sample of health professionals to ensure reliability and validity before data collection. Training was provided to data collectors and the supervisor on the objective of the study, the data collection tool, data collection procedures, and ethical considerations during data collection. The day-to-day supervision was conducted by an assigned supervisor.

### Data quality assurance for qualitative study/trust worthiness of findings

#### Credibility

The investigator took adequate time with the study participants and since the interviewer had previous work experience in a health center, it helped to develop rapport. All interviews were audio recorded and kept for cross-checking, if needed. Peer debriefing and feedback from colleagues and coauthors were used in managing the data. The inquiry process and findings were described in detail so that any reader of the report would be able to use it, and researchers may replicate the study at other similar settings.

#### Transferability

The study participants were selected purposively for KIIs.

The inquiry process and findings were described in detail so that any reader of the report would be able to use it, and researchers may replicate the study at other settings that have similar conditions.

### Measurement

#### Good data quality

The data that fit the criteria for the three quality dimensions are as follows: accuracy ≥80%; completeness ≥85%; and timeliness ≥85% (29, 30).

#### Poor data quality

The data that do not fit the three criteria are as follows: accuracy <80%; completeness <85%; or timeliness <85%.

#### Data accuracy

Data accuracy was measured by calculating the number from the source document over the number from the report submitted to the next level. Based on a 10% tolerance, data accuracy was classified as follows: over-reporting (<0.90% or 90%%); acceptable limit (0.90%–1.10% or 90%–110%); and under-reporting (>1.10% or 110%). The health center data are considered accurate if the average was ≥80% (30).

#### Completeness

Completeness was the average of the source document or registration content completeness and report content. The data were considered complete if the average was ≥85% (31).

#### Timeliness

Timeliness was assessed as a report submission within the accepted time period through observing the reporting date on the reporting form of two randomly selected monthly reports. The data of the health center were considered timely if the average was ≥85% (29).

### Data processing and analysis

For the quantitative study, the data were checked for completeness, coded, and entered in epi-data version 3.1 and the analysis was made using STATA version 14. Bivariate and multivariate binary logistic regression analyses were computed to assess the associations of factors with data quality. The adjusted odds ratio (AOR) with its 95% CI was reported and a *p*-value < 0.05 was considered to be statistically significant. The goodness-of-fit was tested using the Hosmer–Lemeshow statistical test and the *p*-value was >0.05. The qualitative data collected during field visits were organized and coded manually. Finally, a thematic analysis was performed and descriptive summaries were made based on the participants’ descriptions.

### Ethical considerations

Ethical clearance was obtained from the research and ethical review committee of Bahir Dar University College of Medicine and Health Science (Mrf No. BDU/3016/24). A formal written letter was provided to the West Gojjam Zone health office, Woreda health office, and health centers. Participant-related data were kept confidential throughout the study.

## Results

### Sociodemographic characteristics

A total of 304 respondents from 32 health centers were included in the study and the overall response rate was 96.8%. With regard to experience, 171 (56.3%) respondents had less than 5 years of experience (Table 1).

**Table 1:** Sociodemographic characteristics of respondents in health centers of West Gojjam Zone, northwest Ethiopia, 2021 (*N* = 304).

	Frequency (*N*)	Percentage (%)
Age (years)		
<31	146	48.0
≥31	158	52.0
**Experience**		
<5	171	56.3
5–9	81	26.6
10–14	39	12.8
≥15	13	4.3
**Sex**		
Male	142	46.7
Female	162	53.3
**Education level**		
Diploma	107	35.2
Degree	166	54.6
Masters and above	31	10.2
**Job title**		
Nurse	81	26.6
Public health officer	48	15.8
Pharmacy	49	16.1
Midwifery	54	17.4
Laboratory technician	7	2.3
Others	65	21.4
**Others; HIT, doctor, environmental health**		
**Working unit**		
Adult OPD	110	36.2
Dispensary	44	14.5
Maternity	52	17.1
Laboratory	36	11.5
Emergency	8	2.6
HIT room	16	5.3
Under five OPD	9	3
ART room	21	6.9
Immunization room	8	2.6

OPD, out patient department; ART, antiretroviral therapy.

### Factors associated with data quality of the routine health information system

#### Technical factor

Of 304 respondents, 229 (75.33%) agreed that most health information systems require information technology, 218 (71.71%) agreed on the use of both manual paper and computer-based files for recording information, and 213 (70.1%) agreed on the need for trained personnel for data entry (Table 2).

**Table 2:** Technical factors of quality of data at health centers of West Gojjam Zone, northwest Ethiopia, 2021 (*N* = 304).

Technical factor	Frequency (*N*)	Percentage
**Information technology easy to manage**		
Agree	229	75.33
Disagree	75	24.67
**User-friendliness**		
Agree	195	64.14
Disagree	109	35.86
**Complexity of RHIS format makes it hard for health workers to use the system**		
Agree	187	61.51
Disagree	117	38.49
**RHIS application Software scarcity**		
Agree	196	64.47
Disagree	108	35.53
**Training**		
Agree	213	70.1
Disagree	91	29.9
**Mode of data entry**		
Agree	218	71.71
Disagree	86	28.29
**Presence of incomplete data**		
Agree	155	51
Disagree	149	49
**Late data presented**		
Agree	167	54.9
Disagree	137	45.1
**Feedback**		
Agree	104	34.2
Disagree	200	65.8
**Oriented for use of data collection tool**		
Agree	104	34.2
Disagree	200	65.8
**Discussion on monthly performance indicator**		
Agree	167	54.9
Disagree	137	45.1
**Lack of skill in data collection**		
Agree	178	58.55
Disagree	126	41.45
**Lack of skill in data analysis**		
Agree	171	56.25
Dis agree	133	43.75
**Lack of skill of information presentation**		
Agree	174	57.2
Disagree	130	42.8
**Lack of skill to information use**		
Agree	180	59.2
Disagree	124	40.8
Total	304	100

#### Organizational factor

Of 304 respondents, 230 (75.66%) agreed on the lack of sufficient financial resources and 225 (74%) agreed on the staff’s awareness of their responsibilities for data quality of the routine health information systemin the health centers (Table 3).

**Table 3:** Organizational factors of quality of data at health centers of West Gojjam Zone, northwest Ethiopia, 2021 (*N* = 304).

Organizational factor	Frequency (*N*)	Percent (%)
**Organizational rule, value, and practice**		
Agree	219	72.04
Disagree	85	27.96
**Lack of sufficient financial resource**		
Agree	230	75.66
Disagree	74	24.34
**Presence poor leadership and low management support**		
Agree	221	72.70
Disagree	83	27.30
**Routine health information compilation supervision**		
Agree	165	54.28
Disagree	139	45.72
**Able to access to timely report**		
Agree	173	56.9
Disagree	131	43.1
**Gaining timely feedback**		
Agree	136	44.7
Disagree	168	53.3
**Presence of level of culture of information use**		
Agree	218	71.71
Disagree	86	28.29
**Presence of well streamlined RHIS policy**		
Agree	206	67.76
Disagree	98	32.24
**Gaining regular staff meeting to review action plan**		
Agree	183	60.2
Disagree	121	39.8
**Share data with other stakeholders**		
Agree	201	66.1
Disagree	103	33.9
**Staff are aware of their responsibility**		
Agree	225	74
Disagree	79	26
**Staff are trained in data management and use**		
Agree	114	37.5
Disagree	190	62.5
**Report on data accuracy regularly**		
Agree	158	52
Dis agree	146	48
**Use RHIS data for day to day management facility**		
Agree	132	43.4
Disagree	172	56.6
**Gather data to find the root cause of the problem**		
Agree	173	56.9
Disagree	131	43.1
**Use RHIS data for education and community mobilization**		
Agree	193	63.49
Dis agree	111	36.51
Total	304	100

#### Behavioral factor

Of 304 respondents, 237 (74.3%) agreed that data collection is meaningful to individuals, 237 (74.3%) disagreed that data collection makes one bored, and 218 (71.71%) disagreed that collecting information gives the feeling that it is a burden on the individual (Table 4).

**Table 4:** Behavioral factors of quality of data at health centers of West Gojjam Zone, northwest Ethiopia, 2021 (*N* = 304).

Behavioral factors	Frequency (*N*)	Percent (%)
**Knowledge on content of RHIS forms**		
Agree	201	66.12
Disagree	103	33.88
**Problem-solving skill for RHIS tasks**		
Agree	200	65.79
Disagree	104	34.21
**Confidence to use generated information by RHIS management team**		
Agree	204	67.11
Disagree	100	32.89
**Staff competence to perform their RHIS tasks**		
Agree	182	59.9
Disagree	122	40.1
**Staff attitude toward data collection and recording**		
Agree	202	66.4
Disagree	102	36.6
**The belief about Routine RHIS**		
Agree	195	64.1
Disagree	109	35.9
**Lack of motivating incentives to staff during the data collection**		
Agree	208	68.42
Disagree	96	31.58
**Collecting information that adds no value irritates me**		
Agree	87	28.6
Disagree	217	71.4
**Data collection makes one bored**		
Agree	78	25.66
Disagree	226	74.34
**Data collection meaningful to me**		
Agree	237	78
Disagree	67	22
**Collected information used for planning, monitoring**		
Agree	207	68.1
Disagree	97	31.9
**Knowing duties and responsibilities**		
Agree	180	59.2
Disagree	124	40.8
**Collecting information gives a feeling that is a burden on me**		
Agree	86	28.29
Disagree	218	71.71
**Understand and appreciate my roles and responsibilities**		
Agree	145	47.70
Disagree	159	52.30
Total	304	100

### Self-efficacy

**Figure 2:**
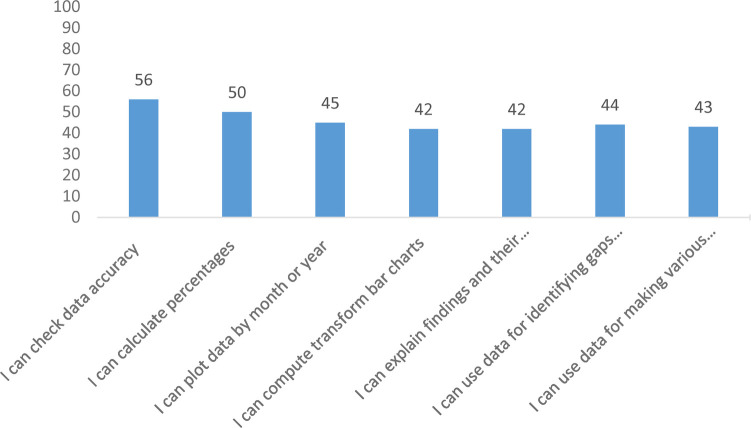
Self-reported level of confidence to perform specific RHIS tasks at health centers of West Gojjam Zone, northwest Ethiopia, 2021.

The confidence level of performing RHIS tasks for health professionals was assessed on a scale of 0–100. The mean score obtained for the seven questions was expressed as a percentage. Higher confidence was observed in checking data accuracy (56%) and lower confidence was observed in explaining findings (42%). The mean confidence level of respondents for performing RHIS activities was 46% (Figure 2).

### Level of data quality

#### Data quality in terms of accuracy

**Figure 3:**
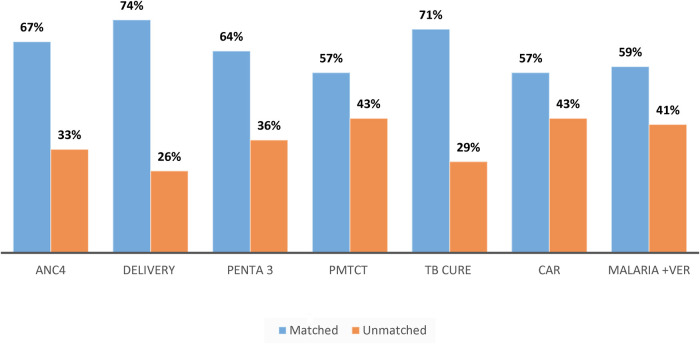
Accuracy of data based on indicator type at health centers of West Gojjam Zone, northwest Ethiopia, 2021.

Among the 32 health centers for which data accuracy was checked, 74% had accurate data while 26% had inaccurate data. The seven data items or indicators were assessed for data accuracy. Service delivery reports and registration books were checked for the months of September and October by random selection of the months. The seven indicators were antenatal care fourth visit (ANC4), contraceptive acceptance rate (CAR), institutional delivery, pentavalent third doses (Penta 3), PMTCT, TB cure rate, and confirmed cases of malaria from top priority indicators at the national level. Data were over-reporting at all health facilities (Figure 3).

#### Data quality in terms of completeness

Content completeness was assessed by checking the 2-month service delivery report and registration content, and whether the required data elements in a report and registration form were filled or data were completed. Based on this, among the 32 health centers for which data completeness was checked, a data element of 70% was registered completely.

#### Data quality in terms of timeliness

Timeliness of the RHIS reports was assessed by checking whether the reported RHIS data by the health centers met the predetermined deadline of the reporting period received by the facility head.

The records of reports received showed that 78% of health center RHIS reports sent met the reporting deadline.

### Bivariate and multivariate logistic regression analysis

Table 5 shows both bivariate and multivariable logistic regression findings. The complexity of the RHIS format, problem-solving skills for RHIS tasks, and known duties, roles, and responsibilities were significantly associated to the data quality in both the bivariate and multivariate analyses. The participants who agreed to the complexity of the RHIS format were 3.8 times more likely to have good data quality compared to those who disagreed to the complexity of the RHIS format (AOR 3.8; 95% CI 1.7–8.50). Those who agreed to problem-solving skills for RHIS tasks were 2.8 times more likely to have good data quality compared to those who disagreed to problem-solving skills for RHIS tasks (AOR 2.8; 95% CI 1.2–6.4). Those who agreed to knowing their duties, roles, and responsibilities were 12 times more likely to have good data quality compared to those who disagreed with knowing their duties, roles, and responsibilities (AOR 12; 95% CI 5.6–25.8) (Table 5).

**Table 5:** Bivariate and multivariate logistic regression analysis of factors associated with data quality at the health center of West Gojjam Zone, northwest Ethiopia, 2021 (*N* = 304).

Variables	Data quality, *n* (%)		COR (95%CI)	AOR (95% CI)	*P*-value
	Good (*N* = 224)	Poor (*N* = 80)			
**Complexity of RHIS**					
Agree	159 (71)	28 (35)	4.5 (2.6–7.8)	3.8 (1.7–8.5)	0.001
Disagree	65 (29)	52 (65)	1	1	
**Problem-solving skill for RHIS tasks**					
Agree	161 (71.9)	39 (48.8)	2.7 (1.6–4.5)	2.8 (1.2–6.4)	0.016
Disagree	63 (28.1)	41 (51.2)	1	1	
**Know duties and roles**					
Agree	165 (73.7)	15 (18.8)	12.1 (6.4–22.9)	12 (5.6–25.8)	0.001
Disagree	59 (26.3)	65 (81.2)	1	1	

## Overall data quality

Based on the three dimensions of data quality, the overall data quality of the health centers was 74%.

## Qualitative result

A total of eight KIIs were conducted. The qualitative finding shows the two following recurring themes: practices of respondents to improve data quality and challenges.

### Practices of respondents to improve data quality

The participants of the KIIs said that the main finding for data quality practices was that there were specific processes dedicated to ensuring the quality of the data. The first way of ensuring data quality practice was by doing a lot of quality assurance sampling. The second way was through a performance monitoring team. The third way was random supervision using an indicator. However, in some health facilities, the performance monitoring team was not working properly and one participant explained it as follows:

We do have any specific things; we do to ensure data quality practice, always by doing LQAS, random supervision, and to some extent, using PMT. (41-year-old female, head of a health center 1)

The data quality assessments were conducted periodically by staff from the health facility and Woreda health office. However, this was not done regularly; when it was done, only a small fraction of the data elements was verified at the health facilities. Most of the key informants explained that, in data quality practice, the staff expects the quality to be verified and ensured by the next level officers at the district during routine data quality assurance visits to the health facility by the district and other subnational level officers.

… in addition, the monthly summary form has to be signed by a superior officer at the health facility verifying the data collected. However, this verification is not usually done, and forms are only signed to allow timely submission to the Woreda health office. (29-year old male key informant, health information technician 2)

### Challenges

Based on the qualitative data, the barriers or challenges of data quality were classified into four categories: clinical work overload; use of complex and bulky forms; poor feedback mechanisms; and lack of generated information at health facilities and inadequate training on health information systems.

The clinical health staff were overburdened with their clinical duties and there was difficulty collecting and managing the data. Therefore, the healthcare workers were already fatigued and did not pay attention to data quality and forgot to manage the data with the routine health information systems at the health facility. Some of the participants raised the issues as follows:

If we have too many patients or on immunization days, we may forget to enter all the patients in the daily registers or only do that after some days or after we have forgotten some of the details. (35-year-old male key informant, health information technician)

Most of the key informants had never had any formal training in the use of the data reporting tools. This poses a serious challenge in ensuring data quality practice.

I wish that I could attend more training on data management but there is no sponsorship or opportunity. (27-year old female key informant, health information technician)

## Discussion

The aim of the present study was to assess the data quality and its associated factors of the routine health information system among health centers in northwest Ethiopia. Data quality in terms of accuracy, completeness, and timeliness was 74%, 78%, and 70%, respectively. The overall data quality of the selected health centers in West Gojjam Zone scored 74%, which was below that of the national acceptable level or target of 90% (32, 33). The findings show poor data quality in health facilities. Poor data quality can lead to inaccurate analysis, poor customer relations, and poor business decisions regarding healthcare and service provision for populations. The reason may be a lack of manpower that performs the data processing at the selected health facilities. The accuracy of data in the health centers of the selected zone was 74%, which is in line with a study conducted in Hadiya Zone (76%) (21). However, this study scored less than a study conducted in Nigeria (79%) (34). It implies that data do not faithfully reflect the actual level of service delivery that was conducted in the selected health facilities. The difference might be because of the difference in the type of facility and the feedback provided to the departments, manpower skills, or data managers. In addition, the interval of verification factor used to measure the data accuracy in Nigeria was wider (0.85–1.15) (34) than the verification factor interval used in this study (0.9–1.1). Data accuracy can be affected by errors that occur during data entry, intentionally manipulating the data for reasons such as competition among staff and facilities, false reports to increase achievement, and reports not finished on time.

Regarding content completeness, the selected district health centers of the zone scored 70%, which is lower than in a study conducted in Mekelle (100%), Hadiya Zone (83.2%), Addis Ababa (96%), and Rwanda (98%) (25, 35–38). This finding implies that there is no sufficient information available when required to make decisions about the health of the population and to target resources to improve health-system coverage, efficiency, and quality in Ethiopia.

This difference may be due to health workers in the present study focusing on managing patients rather than recording data due to the work overload and lack of commitment to the data quality. On the other hand, the results are comparable with a study recently conducted in Harari region (69.6%) and in India (71%) (31, 35). The overall timeliness in the zonal district health centers was scored at 78% based on a 90% tolerance of timeline. In this study, this result of 78% is in line with a study conducted in Hadiya Zone (73%) (21), but higher than a study carried out in Kenya (56%) (39) and lower than in studies conducted in Hadiya Zone (88.4%), Mekelle (100%), and Rwanda (93.85%) (25, 36, 40). This may be due to a difference in the knowledge of respondents about the implications of reporting data in a timely manner and their commitment for data quality. The healthcare workers in the present study may give put less emphasis on data quality rather than focusing only on managing patients.

In this study, the odds of good data quality that was reported by those health workers who agreed to the complexity of the RHIS format is higher than those who disagreed to the complexity of the RHIS format (AOR 3.8; 95% CI 1.7–8.50). This finding implies that the RHIS format is difficult to use for several health professionals and that data quality is compromised. This is supported by the qualitative results in this study and in the quantitative study conducted in Jimma Zone (41). Those participants who agreed to problem-solving skills for RHIS tasks were more likely to have good data quality compared to those who disagreed to problem-solving skills for RHIS tasks (AOR 2.8; 95% CI 1.2–6.4). This was supported by a study conducted in Addis Ababa (42). This might be due to low performance of health workers when engaging in health information-related activities.

According to the qualitative findings of this study, the complexity of registration forms affects data quality, which was supported by studies carried out in Ethiopia, South Africa, and Kenya (43–46). In addition, the lack of skilled human resources appeared to affect all levels of the RHIS process, most prominently at health facilities, where health workers were responsible for data collection on top of their clinical service. This creates a workload for the RHIS. Similar challenges with human resources have been found elsewhere (5, 22, 45). At the health facility level, delays in completing data records have become a typical issue. This does not address the problem of parallel reporting obligations, which added to workload and reporting delays (30). Other qualitative findings, such as lack of training and feedback, affect data quality. This is supported by research conducted in Jimma Zone, Addis Ababa, and Ethiopia as a whole (21, 30, 41). The lack of use of the generated information at health facilities is also another barrier that affects data quality, which is supported by research conducted in Ethiopia (21, 30, 47). This finding has theoretical importance for researchers to study further and practical importance for health policymakers to focus on improving data quality.

### Limitation of study

This study was conducted only at the health center level, which may not be representative of all health facilities. The collected data rely on self-reported exposure to certain factors because the study was not conducted in a longitudinal study design. Since the study design was cross-sectional, it is difficult to establish a causal relationship between the dependent and independent variables.

## Conclusions

Data quality for the three dimensions was scored below the acceptable level of data tolerance. The complexity of the RHIS format, problem-solving skills for RHIS tasks, and knowing one’s duties, role, and responsibilities were significantly associated with data quality in quantitative data; the lack of human resources, use of complex and bulky forms, poor feedback mechanisms, delay in completing data records, and inadequate training on health information systems were barriers affecting data quality. Therefore, all concerned bodies in the Ministry of Health, regional health bureau, and other departments should add more emphasis to improve data quality.

## Data Availability

The original contributions presented in the study are included in the article/Supplementary Material, further inquiries can be directed to the corresponding author.
